# Dipeptidyl-Peptidase IV Activity Is Correlated with Colorectal Cancer Prognosis

**DOI:** 10.1371/journal.pone.0119436

**Published:** 2015-03-19

**Authors:** Gorka Larrinaga, Itxaro Perez, Begoña Sanz, Maider Beitia, Peio Errarte, Ainhoa Fernández, Lorena Blanco, María C. Etxezarraga, Javier Gil, José I. López

**Affiliations:** 1 Department of Nursing I, School of Nursing, University of the Basque Country (UPV/EHU), Leioa, Bizkaia, Spain; 2 Department of Physiology, Faculty of Medicine and Dentistry,University of the Basque Country (UPV/EHU), Leioa, Bizkaia, Spain; 3 Department of Anatomic Pathology, Basurto University Hospital,University of the Basque Country (UPV/EHU), Bilbao, Bizkaia, Spain; 4 Department of Anatomic Pathology, Cruces University Hospital, University of the Basque Country (UPV/EHU), Barakaldo, Bizkaia, Spain; 5 BioCruces Health Research Institute, Barakaldo, Bizkaia, Spain; Sapporo Medical University, JAPAN

## Abstract

**Background:**

Dipeptidyl-peptidase IV (EC 3.4.14.5) (DPPIV) is a serine peptidase involved in cell differentiation, adhesion, immune modulation and apoptosis, functions that control neoplastic transformation. Previous studies have demonstrated altered expression and activity of tissue and circulating DPPIV in several cancers and proposed its potential usefulness for early diagnosis in colorectal cancer (CRC).

**Methods and principal findings:**

The activity and mRNA and protein expression of DPPIV was prospectively analyzed in adenocarcinomas, adenomas, uninvolved colorectal mucosa and plasma from 116 CRC patients by fluorimetric, quantitative RT-PCR and immunohistochemical methods. Results were correlated with the most important classic pathological data related to aggressiveness and with 5-year survival rates. Results showed that: 1) mRNA levels and activity of DPPIV increased in colorectal neoplasms (Kruskal-Wallis test, p<0.01); 2) Both adenomas and CRCs displayed positive cytoplasmic immunostaining with luminal membrane reinforcement; 3) Plasmatic DPPIV activity was lower in CRC patients than in healthy subjects (Mann-U test, p<0.01); 4) Plasmatic DPPIV activity was associated with worse overall and disease-free survivals (log-rank p<0.01, Cox analysis p<0.01).

**Conclusion/significance:**

1) Up-regulation of DPPIV in colorectal tumors suggests a role for this enzyme in the neoplastic transformation of colorectal tissues. This finding opens the possibility for new therapeutic targets in these patients. 2) Plasmatic DPPIV is an independent prognostic factor in survival of CRC patients. The determination of DPPIV activity levels in the plasma may be a safe, minimally invasive and inexpensive way to define the aggressiveness of CRC in daily practice.

## Introduction

CRC is the third commonest malignancy in males and females in the United States, with more than 136,500 estimated new cases and more than 50,000 estimated deaths in 2014 [1]. Europe and other developed regions show similar rates of incidence and mortality [2]. Traditionally related to dietary habits [3], its frequency has steadily increased in the last decades in Western countries as a result of modern style of life to become a health problem of major concern. Huge resources are being invested in prevention and early diagnosis of this disease. Population-based screening campaigns try to discover as much early tumors and precursor lesions as possible, aiming to decrease the incidence of the disease, to simplify the clinical management of patients once the lesion develops, and to improve survival.

From a pathological perspective, adenomatous lesions in the large bowel are fully accepted precursors of CRC [4,5] and the adenoma-carcinoma sequence still provides a solid model for research on carcinogenesis [6]. However, a large number of cellular metabolic processes still largely unknown are involved in the origin and development of neoplastic processes [7].

Peptidases play a key role in carcinogenesis in several ways, for instance regulating bioactive peptides that are crucial in neoplastic growth and immune response, degrading the extracellular matrix, acting as adhesion molecules or participating in intracellular signalling [8]. Besides, some studies have demonstrated that the activity and expression of these enzymes vary in different tumors depending on the several pathological parameters like histological Grade, Stage, and patient survival [8–12]. For this reason, peptidases are useful tools in the development of clinical strategies for treatment and follow up of cancer patients.

Dipeptidyl-peptidase IV (EC 3.4.14.5) (DPPIV), also known as cluster differentiation antigen CD26, is a serine peptidase expressed on a variety of cells including epithelial, endothelial and lymphocytes [13]. As other membrane-bound glycoproteins, it also can be released in active form to body fluids such as plasma, serum and urine [14]. DPPIV cleaves N-terminal dipeptides from selected bioactive peptides including some cytokines, chemokines and neuropeptides, leading to their inactivation and/or degradation. Independent of its enzymatic activity, DPPIV interacts with extracellular matrix (ECM) components including collagen and fibronectin, thus regulating cell-cell and cell-ECM interactions, and with several receptors and proteases that leads to the secretion of matrix metalloproteases (MMPs). Through these multiple functions, DPPIV regulates diverse biological processes including cell differentiation, adhesion, immune modulation and apoptosis, functions that control neoplastic transformation [13].

Many studies have described that DPPIV expression and activity is significantly altered in several solid tumor tissues and that these changes are associated with tumor grade, stage and patients 5-year survival, which point to this enzyme as a potential diagnostic tool and therapeutic target [12,13,15]. Moreover, it has been demonstrated that blood DPPIV levels are significantly lower in cancer patients than in healthy individuals, a finding that has become of great interest in the development of additional tumor markers [14,16–19].

Regarding CRC, two recent studies described that higher immunohistochemical expression of DPPIV in CRC tissues is correlated with metastases and worse overall survival of CRC patients [20] and that circulating DPPIV levels are correlated with distant recurrence of CRC [21]. We and other groups also described that the expression and activity profile of other serine peptidases related with DPPIV vary throughout the adenoma-adenocarcinoma sequence and that are independently correlated with survival of CRC patients [22–26]. All these accumulated evidences point to the analyses of serine peptidases such as DPPIV as promising tools in the design of new diagnostic/prognostic biomarkers and therapeutic targets in CRC [14,20–26].

In this context, the objective of the present work was to study in a prospective way the metabolic and expression profiles of DPPIV in patients with CRC analyzing tissue from the adenoma-adenocarcinoma sequence and plasma samples, and to correlate the obtained results with classic histopathological parameters for tumor prognosis and survival.

## Materials & Methods

The authors declare that all experiments carried out in this study comply with current Spanish and European Union legal regulations. Samples and data from patients included in this study were provided by the Basque Biobank for Research-OEHUN (www.biobancovasco.org). All patients were informed about the potential use for research of their surgically resected tissues, and accepted this eventuality by signing a specific document approved by the Ethical and Scientific Committees of the Basque Country Public Health System (Osakidetza) (CEIC 11/51).

### Patients

A total of 116 patients with CRC were prospectively included in the study. All patients received partial colectomies. The topographic distribution was 41 right sided CRCs, 51 left sided and 24 from the rectum. 7 patients received preoperative therapy, 49 patients received chemotherapy or chemo-radiotherapy after resection, and 60 patients did not receive any therapy.

In 20 patients, both adenomatous and adenocarcinomatous polyps were diagnosed. 13 adenomas were tubular, 6 were tubulovillous adenomas, and 1 villous adenoma. In addition, 16 showed mild to moderate dysplasia and 4 showed high grade dysplasia. The average size was 2.1cm (with a range of 0.6–4cm). These 20 cases were used to analyze the DPPIV activity and mRNA/protein expression throughout the normal mucosa-adenoma-adenocarcinoma sequence.

The American Joint Committee on Cancer system (AJCC, 7^th^ edition) [27] has been applied to assign Stage and Grade. Follow-up was closed by June 30, 2014. At that time, 43 patients (37%) had died of disease. Mean follow-up was 53 months (range 4–88).

Plasma was also collected preoperatively and analyzed in 98 of these patients. Plasma from 72 healthy volunteers with no clinical history of neoplastic diseases was used as control sample.

Clinical data included in the study were retrieved from the patient clinical records and are summarized in Table 1.

**Table 1:** Clinical and pathologic characteristics of patients with colorectal cancer (CRC), both for CRC tissue and plasmatic samples.

Variables	CRC tissue n = 116	Plasma n = 98
**Age**		
Average	70	68
Range	32–89	32–85
**Sex**		
Male	72	63
Female	44	35
**Grade**		
Low (G1–G2)	95	81
High (G3–G4)	21	17
**Local invasion**		
Low (T1–T2)	18	25
High (T3–T4)	98	73
**Nodal invasion**		
No	76	73
Yes	40	25
**Distant Metastases**		
No	106	89
Yes	10	9
**Lymphatic invasion**		
No	103	84
Yes	13	14
**Vascular invasion**		
No	95	80
Yes	21	18
**Perineural invasion**		
No	110	94
Yes	6	4
**Grouped stage**		
Low (0-IIC)	71	70
High (IIIA-IV)	45	28

### Tissue Specimens

Surgical resections were submitted *in fresh* to the Pathology Lab within a period of 30 minutes after removal. The material for this study comes from the excess tissue of pathological diagnosis. Handling of specimens was performed following conventional protocols for the management of surgical resections of colon and rectum [28]. Tumor characteristics were recognized on gross examination and selected fragments of tumor and non tumor tissues were frozen in isopentane and stored at -80°C. Routine procedures in the Pathology Lab included formalin fixation of the surgical specimen and paraffin embedding of the tissue fragments selected for histopathological examination. Histological slides were stained with hematoxylin-eosin. Besides, peripheral venous blood samples from 98 of these patients were obtained prior to surgery and centrifuged at 1500 rpm during 15 minutes. The obtained plasma was also stored at -80°C. Enzyme assay was been performed in plasma obtained from 72 healthy volunteers (matched by sex and age).

### Sample preparation

Explanted tumor samples were homogenized in 10 mM Tris-HCl buffer at pH 7.4, for 30 seconds at 800 rpm using a Heidolph PZR 50 Selecta homogenizer, and ultracentrifuged in a Centrikon T-2070 Kontron Instruments apparatus at 100,000 *g* for 35 minutes. To avoid contamination with soluble enzymes, the resulting pellets were washed three times by suspension in 10 mM Tris-HCl buffer at pH 7.4. Pellets were then homogenized in 10 mM Tris-HCl buffer at pH 7.4, and centrifuged at low speed (1500 *g*) for 3 min to purify the samples. The supernatants thus obtained were used to determine DPPIV activity and protein concentrations. All the aforementioned steps were carried out at 4°C.

### DPPIV activity measurements

DPP IV activity was measured in triplicate by using H-Gly-Pro-β-naphthylamide as substrate, following a modified version of the method of Alponti et al. [29]. The assay is based on the fluorescence of products generated from the hydrolysis of the substrate by the enzyme. Reactions were initiated by adding 30–50 μL of sample to 1 mL of the appropriate incubation mixture (50 mM Tris-HCl buffer, pH 7.4, and 0.2 mM aminoacyl-β-naphthylamide). After 30 min incubation at 37°C, 1 mL of 0.1 M sodium acetate buffer (pH 4.2) was added to the mixture to terminate the reaction. The released product was determined by measuring the fluorescent intensity with a Shimadzu RF-540 Spectrofluorophotometer (the excitation and emission wavelengths were 345 and 412 nm, respectively). Blanks were used to determine background fluorescence. The relative fluorescence was converted into picomoles of product using a standard curve constructed with increasing concentrations of β-naphthylamine.

To verify that the formation of β-naphthylamine (β-NA) was due to the action of DPPIV and not due to other enzymes, we performed inhibition assays in CRC tissue and plasma with a specific inhibitor of the enzyme (diprotin A, 0.2mM). The releasing of β-NA was mainly inhibited in colorectal tissues (82%) and in plasma samples (86%).

Protein concentration was measured in triplicate by the Bradford method [30], using BSA (1 mg/mL) as the calibrator. Results from the CRC tissues and from plasma samples were recorded as units of peptidase per milligram of protein (UP/mg prot) and per liter of plasma (UP/L), respectively. One unit of peptidase activity (UP) is the amount of enzyme required to release one pmol of β-naphthylamine per minute. Fluorogenic assays were linear with respect to hydrolysis time and protein content.

### Immunohistochemistry

Formalin-fixed and paraffin-embedded tissue from 20 CRCs, adenomatous polyps and the uninvolved surrounding mucosa were immunostained with a monoclonal antibody specific for DPPIV/CD26 (Novus Biologicals NB 100–59021, rabbit anti-human, working dilution 1:250). The immunostaining process was performed following routine methods in an automatic immunostainer (Dako Autostainer Plus). In short, endogenous peroxidase activity was blocked by incubating the slides in 3% hydrogen peroxide in absolute methanol for 10 minutes. Antigen retrieval was carried out in citrate buffer (10 mM, pH = 6) for 15 minutes at 100°C in a microwave oven. The primary antibody was applied for 1 hour at room temperature. A subsequent reaction was performed with secondary antibodies and biotin-free HRP enzyme labeled polymer of the EnVision-Flex detection system (Dako, Carpinteria, CA). Nonspecific IgG was used as a negative control. A positive reaction was visualized with diaminobenzydine solution followed by counterstaining with haematoxylin.

### Real-time quantitative RT-PCR Analysis

The expression of *DPP4*, the gene that encode DPPIV, was analysed in tissue from 20 CRCs, adenomatous polyps and the uninvolved surrounding mucosa. To avoid degradation, tissue sections were immersed in RNAlater immediately after surgery and stored at -80°C until processed. Total RNA was extracted from 20 mg of tissue using TriReagent (Sigma, St. Louis, MO). The RNA samples were then incubated with RNase-free DNase I and RNasin (Promega, Madison, WI) to remove residual genomic DNA. First-strand cDNA was synthesized from 5 μg of total RNA of each human sample using First-strand cDNA synthesis kit (Roche, Mannheim, Germany). The resulting cDNA samples were amplified by PCR with specific oligonucleotide primer pairs designed with the analysis software Primer 3 and synthesised by Sigma-Genosys (Cambridge, UK). Based on previous experiments on human renal cell carcinoma [15], CRC [12] and other human tissues [31] we chose glyceraldehyde-3-phosphate dehydrogenase (*GAPDH*), polymerase (RNA) II (DNA-directed) polypeptide A (*POLR2A*), hypoxanthine phosphoribosyltransferase 1 (*HPRT1*), β-actin (*ACTB*), succinate dehydrogenase complex, subunit A (*SDHA*), TATA box binding protein (*TBP*) and peptidylprolyl isomerase A (*PPIA*) as endogenous reference genes. The sequence of the primers used to amplify *DPP4* and the seven housekeeping genes are shown in Table 2.

**Table 2:** Sequence of forward (F) and reverse primers (R) of indicated target genes and the size expected for each PCR-amplified product.

Peptidase	Gene symbol	Fordward primer	Reverse primer	Amplicon size (bp)
DPPIV	*DPP4*	5′-AGTGGCGTGTTCAAGTGTGG-3′	5′-CAAGGTTGTCTTCTGGAGTTGG-3′	112
**Housekeeping Gene name**				
Glyceraldehyde-3-phosphate dehydrogenase	*GAPDH*	5′-CAATGCCTCCTGCACCAC-3′	5′-CCTGCTTCACCACCTTCTTG-3′	350
Polymerase (RNA) II (DNA directed) polypeptide A	*POLR2A*	5′-ACATCACTCGCCTCTTCTACTCC-3′	5′-GTCTTGTCTCGGGCATCGT-3′	268
Hypoxanthine phosphoribosyltrans-ferase 1	*HPRT1*	5′-GCCAGACTTTGTTGGATTTGA-3′	5′-GGCTTTGTATTTTGCTTTTCC-3′	130
Beta-actin	*ACTB*	5′-TCCCTGGAGAAGAGCTACGA-3′	5′-ATCTGCTGGAAGGTGGACAG-3′	362
Succinate dehydrogenase complex, subunit A	*SDHA*	5′-TCTGCCCACACCAGCACT-3′	5′-CCTCTCCACGACATCCTTCC-3′	142
TATA box binding protein	*TBP*	5′-GGATAAGAGAGCCACGAACCAC-3′	5′-TTAGCTGGAAAACCCAACTTCTG-3′	139
Peptidylpropyl isomerase A	*PPIA*	5′-GGTCCCAAAGACAGCAGAAAA-3′	5′-TCACCACCCTGACACATAAACC-3′	114

Primers for the assayed housekeeping genes are also shown.

The expression of *DPP4* and the housekeeping genes was quantified in all cDNAs by real-time PCR using the iCycler iQ real-time detection apparatus (BioRad Laboratories, Hercules, CA, USA). Experiments were carried out essentially as described previously [12,15,32]. Dilutions of the cDNA template were prepared from each tissue and amplified in triplicate using SensiFAST SYBR Mix (Bioline Ltd., London, UK). Three negative controls (with no template, no reverse transcriptase and no RNA in the reverse transcriptase reaction) were also included in each plate to detect any possible contamination. After a hot start (10 min at 94°C), the parameters used for PCR amplification were: 10 s at 94°C, 20 s at 60°C and 30 s at 72°C, for 50 cycles.

Real-time PCR data were expressed as the fold change of the target gene expression relative to the geometric mean (g.m.) mRNA expression of the housekeeping genes in each sample, as described by Vandesompele et al. [33]. The fold change in gene expression was calculated by the formula: 2^-ΔΔ C^_T_, where C_T_ is the threshold cycle, calculated by the iCycler software, ΔC_T_ = (C_Ttarget gene_-C_Tg.m.reference genes_) and ΔΔC_T_ = (ΔC_Ttest sample_-ΔC_Tcontrol sample_).

Samples from the same patient were always measured in the same analytical run to exclude between-run variations. PCR data obtained in one of the normal CRC samples were arbitrarily chosen as control, and this sample was included in all PCR experiments to correct for possible interassay variations.

### Statistical analysis

Kolmogorov-Smirnov and Shapiro-Wilk tests were applied to data obtained from tissue and plasma samples respectively to know if the numbers followed or not a normal distribution. Based on this information (p<0.05), DPPIV activity in tissue and plasma was analyzed with non parametric probes: Mann-Whitney test and Kruskal-Wallis tests were used to detect differences between two or three groups, respectively.

Finally, Kaplan-Meier curves and log-rank test were performed to evaluate the association between DPPIV activity and 5-year survival, comparing groups created by cut-off points based on receiver-operating characteristic (ROC) curves. A Cox regression model was used to test the independent effects of clinical and pathological variables and DPPIV activity on survival. SPSS® 21.0 software was used for the statistical analysis.

## Results

### DPPIV activity and expression in colorectal tissues from CRC patients

When DPPIV activity was analyzed throughout the colorectal adenoma-adenocarcinoma sequence, higher activity was found in CRCs and adenomas than in the adjacent uninvolved mucosa (Kruskal-Wallis test, p = 0.015). *DPP4* mRNA expression showed a similar pattern, with higher levels in both neoplasms than in the non tumor tissue (Kruskal-Wallis test, p = 0.001). These results are described in Table 3.

**Table 3:** 

(A) DPPIV activity	UP/ mg prot (Media ± S.E)	Mann-Whitney (p =)
**Adenocarcinoma**	2713 ± 627	0.806^a^
**Adenoma**	2982 ± 659	**0.007^b^**
**Uninvolved mucosa**	990 ± 139	**0.027^c^**

(B) *DPP4*	Relative Units	Mann-Whitney (p =)
**Adenocarcinoma**	241 ± 66	0.594^a^
**Adenoma**	199 ± 35	**0.021^b^**
**Uninvolved mucosa**	43 ± 14	**0.001^c^**

**(A)** DPPIV/CD26 activity in colorectal tissues from CRC patients. Values are means ± SE of peptidase activity recorded as pmol of units of peptidase (UP) per milligram of protein. Differences were statistically significant (Kruskal-wallis test, p = 0.015). **(B)** *DPP4* gene expression in colorectal tissues from CRC patients. qRT-PCR data for each analyzed sample are recorded as relative units (see text for details). Differences were statistically significant (Kruskal-wallis test, p = 0.001).

^(a)^Adenocarcinoma vs adenoma;

^(b)^Adenocarcinoma vs uninvolved mucosa;

^(c)^Adenoma vs uninvolved mucosa.

When data were stratified by topographic distribution of CRCs, we did not found any significant difference (Kruskal-Wallis test, p = 0.965). Tissue DPPIV activity was also similar between patients who received preoperative treatment, postoperative therapy and patients who did not received any therapy after resection (Kruskal-Wallis test, p = 0.842).

**Fig 1:**
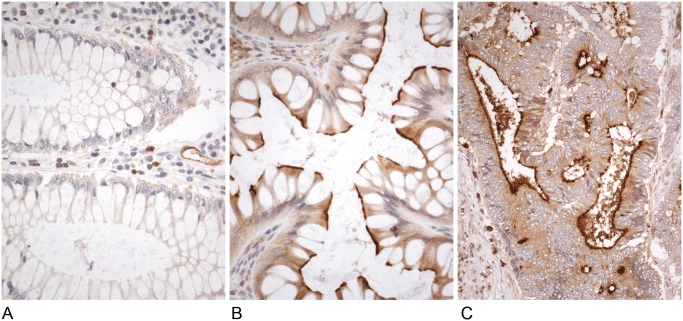
Immunohistochemical expression of DPPIV in normal colorectal mucosa (A), tubulovillous colonic adenoma (B) and colorectal adenocarcinoma (C). DPPIV immunostaining is located in the cytoplasm of adenomas and CRC cells displaying luminal membrane reinforcement.

Fig. 1 shows the immunohistochemical expression of DPPIV in colonic adenocarcinoma, adenoma and normal adjacent mucosa. Normal mucosa did not express DPPIV immunostaining. However, both adenomas (tubular, tubulovillous and villous) and CRCs displayed positive cytoplasmic immunostaining with luminal membrane reinforcement. The inflammatory component in all cases was also positive.

### Tissue DPPIV activity according to 5-year survival and to pathologic characteristics

**Fig 2:**
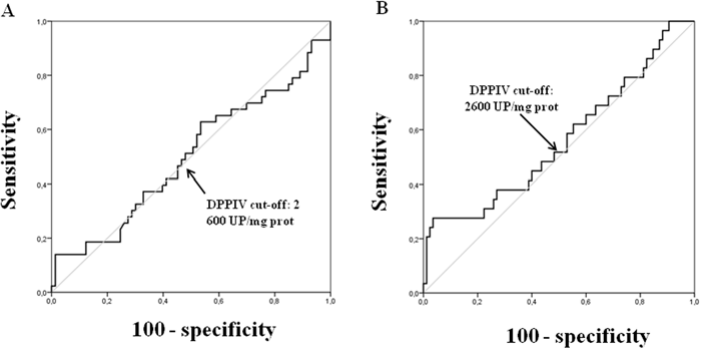
ROC curves for tissue DPPIV activity. Optimal sensitivity and specificity ratios were observed using the following cut-off value: 2600 UP/mg prot of tissue DPPIV activity for OS (**A**) and DFS (**B**).

To determine the best cut-off values for overall survival (OS) (Fig. 2A) and disease-free survival analyses (DFS) (Fig. 2B), ROC curves were performed. For tissue DPPIV activity, 2600 UP/mg protein cut-off point showed the most optimal sensitivity and specificity ratios (Se = 47% and Sp = 55% for OS, and Se = 48% and Sp = 55% for DFS) (Fig. 2A and 2B).

**Fig 3:**
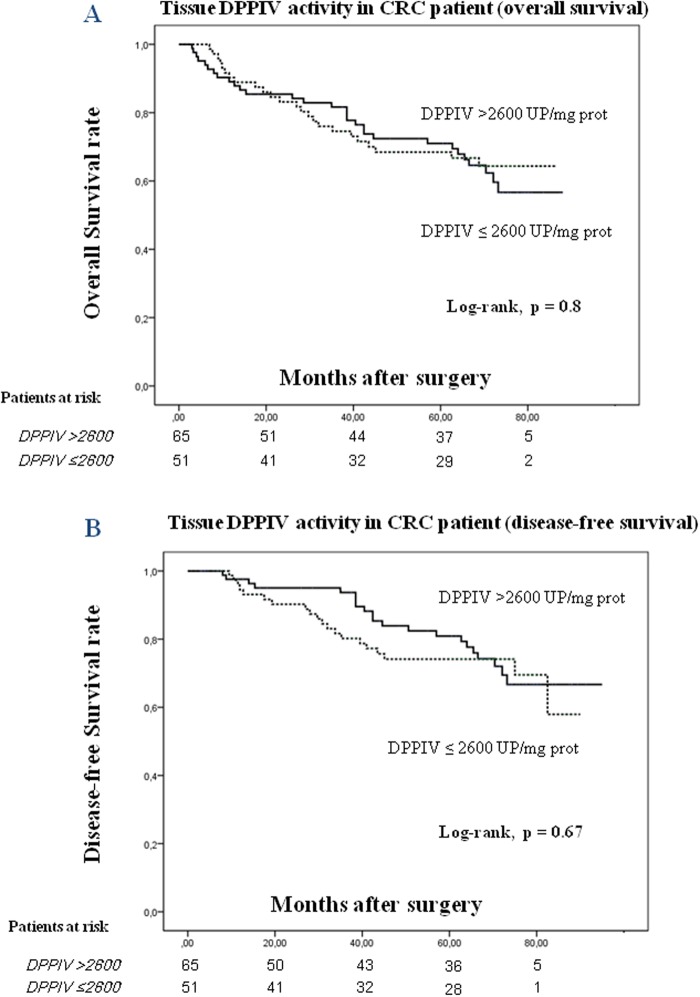
Kaplan-Meier curves for DPPIV activity in CRC tissues. Overall survival (**A**) and disease-free survival curves (**B**) of CRC patients according to their tumor DPPIV activity pattern. The number of patients at risk in each group at individual time points is also included.

Kaplan-Meier curves and log-rank test showed that five-year OS and DFS of patients with CRC was not correlated with tissue DPPIV activity (log-rank test, p = 0.8 for OS; and p = 0.67 for DFS) (Fig. 3A and 3B, respectively).

Similarly, the stratification of DPPIV activity by several pathologic variables did not throw any statistically significant result (Table 4).

**Table 4:** DPPIV/CD26 activity in CRC tissue according to pathologic characteristics.

Variables	UP/mg prot (Media ± S.E)	Mann-Whitney (p =)
**Grade**		
Low (G1–G2)	3357 ± 408	0.648
High (G3–G4)	2959 ± 673	
**Local invasion**		
Low (T1–T2)	1750 ± 292	0.061
High (T3–T4)	3506 ± 396	
**Nodal invasion**		
No	3221 ± 415	0.821
Yes	3376 ± 643	
**Distant Metastases**		
No	3082 ± 315	0.523
Yes	5295 ± 1212	
**Lymphatic invasion**		
No	3046 ± 298	0.742
Yes	5332 ± 1245	
**Vascular invasion**		
No	2935 ± 329	0.128
Yes	4856 ± 1217	
**Perineural invasion**		
No	3225 ± 366	0.135
Yes	4317 ± 1239	
**Grouped stage**		
Low (0-IIC)	3022 ± 375	0.532
High (IIIA-IV)	3615 ± 651	

Values are means ± SE of peptidase activity recorded as pmol of units of peptidase (UP) per milligram of protein.

### DPPIV activity in plasma samples from CRC patients

**Fig 4:**
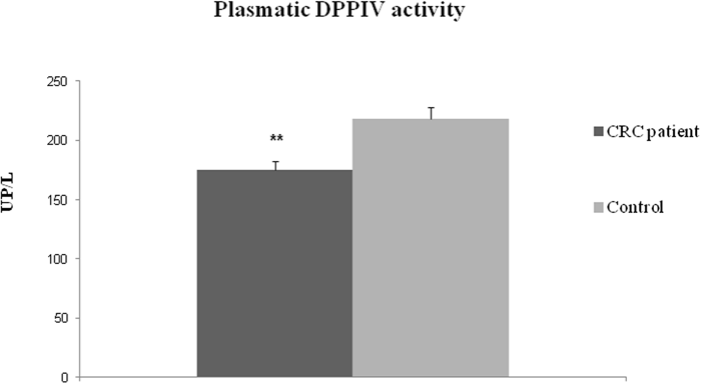
Plasmatic DPPIV activity in CRC patients and healthy subjects (controls). Values are means ± SE of units of peptidase per liter of plasma (UP/L). (*) Student’s T test, p<0.01.

In plasma from CRC patients DPPIV activity was significantly lower than in healthy individuals (175 ± 7.2 UP/L vs. 218 ± 10.1 UP/L, Mann-Whitney test p<0.01) (Fig. 4). However the stratification of data according to pathological variables did not yield any significant result (Table 5).

**Table 5:** DPPIV/CD26 activity in plasma from CRC patients according to pathologic characteristics.

Variables	UP/L (Media ± S.E)	Mann-Whitney (p =)
**Grade**		
Low (G1–G2)	176 ± 11	0.053
High (G3–G4)	124 ± 26	
**Stage**		
Low (T1–T2)	200 ± 28	0.271
High (T3–T4)	157 ± 11	
**Nodal invasion**		
No	171 ± 13	0.633
Yes	157 ± 21	
**Distant Metastases**		
No	166 ± 11	0.939
Yes	178 ± 42	
**Lymphatic invasion**		
No	166 ± 11	0.985
Yes	170 ± 34	
**Vascular invasion**		
No	169 ± 12	0.774
Yes	158 ± 23	
**Perineural invasion**		
No	165 ± 11	0.352
Yes	195 ± 29	
**Grouped stage**		
Low (0-IIC)	171 ± 13	0.535
High (IIIA-IV)	155 ± 19	

Values are means ± SE of units of peptidase per liter of plasma (UP/L).

**Fig 5:**
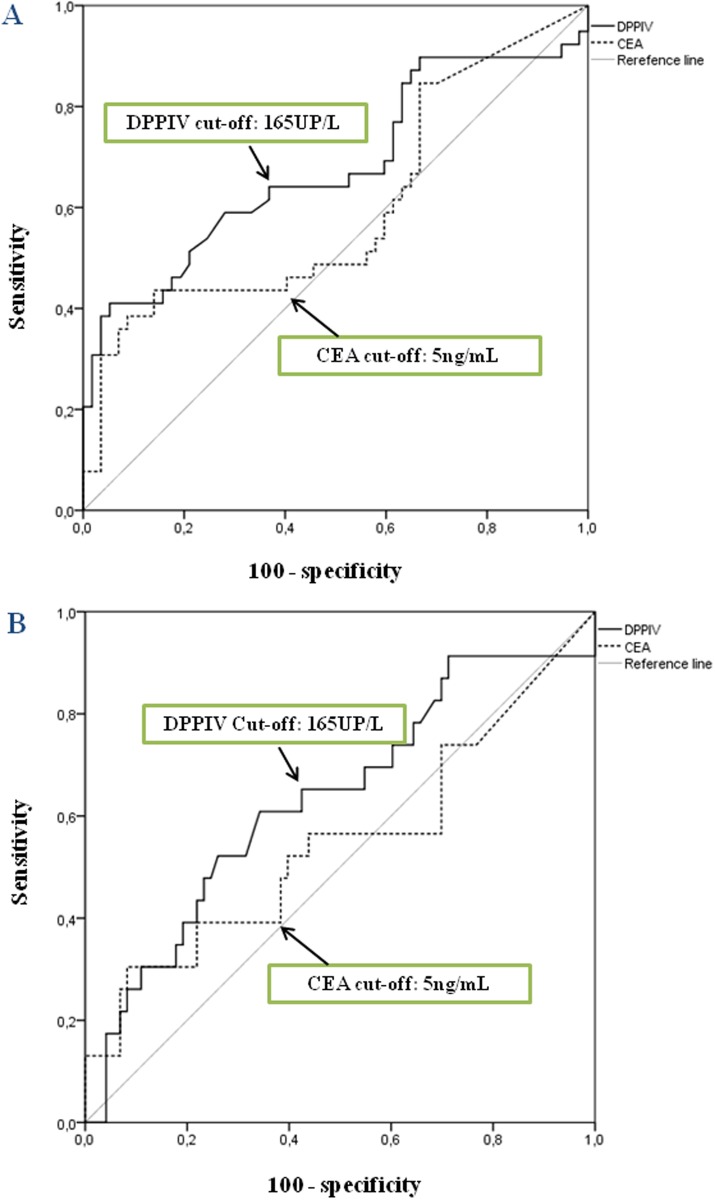
ROC curves for plasmatic DPPIV activity and CEA levels. Optimal sensitivity and specificity ratios were observed using the following cut-off value: 165 UP/L for plasmatic DPPIV activity both for OS (**A**) and DFS (**B**). A cut-off value of 5 ng/mL for CEA, showed lower sensitivity but higher specificity than DPPIV.

In plasma samples ROC curves were performed both for DPPIV activity and carcinoembryonic antigen (CEA) levels. For plasmatic DPPIV activity, 165 UP/L cut-off value showed the most optimal sensitivity and specificity ratios (Se = 64% and Sp = 63% for OS, and Se = 65% and Sp = 58% for DFS) (Fig. 5A and 5B). A cut-off value of 5 ng/mL for CEA, which is known to have a prognostic impact in CRC [34], showed lower sensitivity (44% for OS, and 39% for DFS) but higher specificity (68% for OS, and 64% for DFS) than DPPIV (Fig. 5A and 5B).

**Fig 6:**
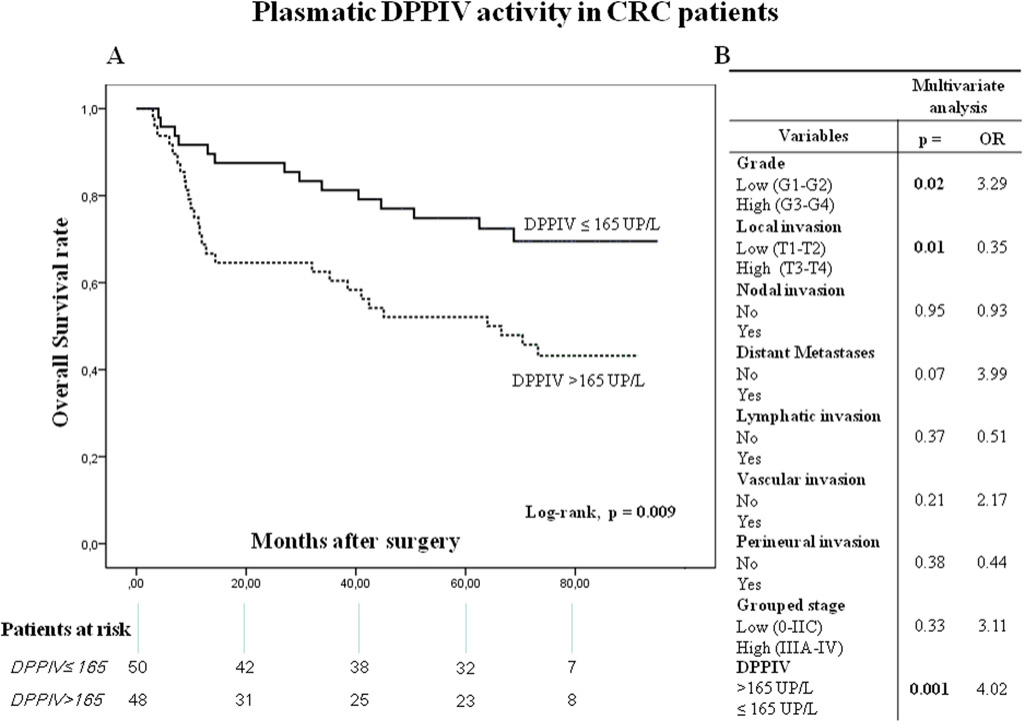
Overall survival (OS) of CRC patients according to their plasmatic DPPIV activity pattern. (**A**) Kaplan-Meier curve and log-rank test. (**B**) Multivariate analysis of clinicopathologic variables and plasmatic DPPIV activity in predicting OS. The number of patients at risk in each group at individual time points is also included.

**Fig 7:**
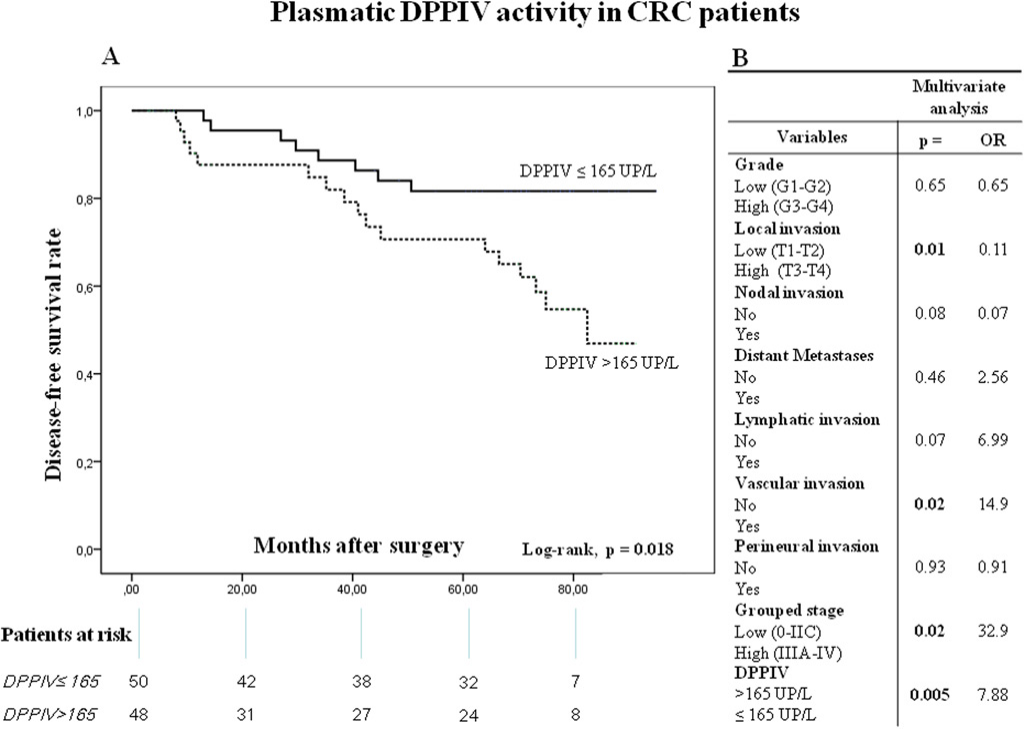
Disease-free survival (DFS) of CRC patients according to their plasmatic DPPIV activity pattern. (**A**) Kaplan-Meier curve and log-rank test. (**B**) Multivariate analysis of clinicopathologic variables and plasmatic DPPIV activity in predicting DFS. The number of patients at risk in each group at individual time points is also included.

Kaplan-Meier curves and log-rank test showed that both OS (Fig. 6) and DFS (Fig. 7) were inversely correlated with plasmatic DPPIV activity. Thus, when plasmatic DPPIV activity was higher than 165 UP/L the OS and DFS were significantly worse (log-rank p = 0.009 and = 0.018, respectively) (Figs. 6A and 7A).

On the other hand, multivariate analysis showed that plasmatic DPPIV activity (p = 0.001), histological grade (p = 0.02) and local invasion (p = 0.01) were independent factors influencing patient OS (Fig. 6B), and that plasmatic DPPIV activity (p = 0.005), vascular invasion (p = 0.02) and grouped stage (p = 0.02) were independent prognostic factors of DFS (Fig. 7B).

## Discussion

Malignant transformation from normal to cancerous tissue is associated with cell-surface glycoprotein modifications. These phenotypic changes may play a crucial role in oncogenesis and can be useful tumor markers in distinguishing malignant from benign tissues [8]. Regarding CRC, the adenoma-carcinoma sequence in the large bowel describes that the gradual progression from normal to dysplastic epithelium, and hence to carcinoma, is the result of the successive accumulation of genetic mutations that lead to altered levels of several glycoproteins, like some peptidases [6,8].

The first relevant result in our study was that DPPIV showed higher activity and mRNA levels in preneoplastic adenomatous lesions and in CRC when compared with the uninvolved surrounding mucosa, although it did not correlate with pathological variables and with 5-year survival of CRC patients. Previously, it was reported that the activity and expression of two other serine peptidases, fibroblast activation protein alpha (FAP) and prolyl endopeptidase (PEP), was higher in adenomas and in early stages of CRC respectively [22,24]. In addition, FAP expression correlated with worse survival [22,23] and PEP activity did it with a better overall and disease-free survival [25]. Although these divergent influences on CRC prognosis suggest different acting roles for these serine peptidases in this disease, the expression and activity increases of these peptidases in neoplastic and preneoplastic tissues should be taken into account when designing new therapeutic approaches for colorectal cancer.

Due to its variable expression on solid tumors and to its diverse biological functions, the exact role that DPPIV plays in cancer remains to be elucidated. It has been described that DPPIV may either promote or impede tumor development depending on the specific type of tumor or on the phase of development the tumor is [13]. This phenomenon of tumor specificity sets forth practical difficulties when applying peptidase inhibitors in cancer therapies, and this point is being actively investigated nowadays [35,36]. A new trend in this topic consists in the use of cytotoxic prodrugs enabled to be activated by specific peptidases only where these peptidases are more active or highly expressed, aiming to damage the neoplastic cell without modifying the enzyme functionality [35,36]. Some investigators are working in prodrugs with FAP as a target [35]. Since its homologous DPPIV is also highly active in adenomas and CRC, we suggest that this serine peptidase could be the target of similar prodrugs.

Cell-surface glycoproteins can be released to the extracellular space, appear in diverse body fluids and have become useful serum or plasmatic biomarkers for helping in screening, diagnosis, staging, prognosis and monitoring cancer therapy. The most relevant example in the clinical following of CRC patients is the carcinoembryonic antigen (CEA) [34]. However, the analysis of DPPIV in the plasma or serum of these patients has proved to be a reliable method in the early detection of CRC, being complementary to the classic faecal occult blood exam and other serum biomarkers which are under clinical investigation nowadays [14,17–20].

Several authors [14,17,19] have described decreases in the circulating levels of DPPIV of CRC patients compared with healthy subjects. In addition, increases of this enzyme have been observed in the serum of metastatic *vs*. non metastatic patients [14,17,19,20]. These data were obtained by immunodetection, suggesting its potential usefulness for early diagnosis and clinical follow-up. We also detected that the activity of DPPIV measured by fluorimetric methods was significantly decreased in the plasma of CRC patients. By contrast, we found that this activity was not correlated with metastatic status or with any other pathological variable.

The high differences in the number of patients between the groups with and without metastases could be on the origin of this partial discrepancy. Other causes should also be considered because it has been described that several biological phenomena may influence the activity of circulating DPPIV/CD26 without affecting the protein level. In this sense the presence of circulating molecules that alter the activity of this enzyme has been reported [37,38]. Nazarian et al. [38] have shown in a very recent study that metastatic prostate cancer patients presented decreased circulating DPPIV activity while protein levels were similar with respect to patients with localized primary tumor. This finding pointed to a circulating small peptide as a possible cause of this inhibition [38] and opens new perspectives for further studies to clarify the potential usefulness of the combined analysis of DPPIV activity and protein levels in the serum/plasma of cancer patients.

Since plasmatic levels and activities of the PEP and FAP have also been correlated with the survival of CRC patients [19,25], a main objective of our study was to analyze prospectively the correlation among plasmatic activity of its homologous DPPIV and 5-year survival of the patients. Data showed that patients with higher plasmatic DPPIV activities had poorer overall and disease-free survival. These data showed better sensitivity but worse specificity than those obtained with CEA. The multivariate analysis demonstrated that DPPIV is an independent prognostic factor influencing CRC patient survival. Therefore, there are convincing evidences that the determination of circulating DPPIV may be helpful in the early diagnosis [14,17–19,21] and also in the prognosis of CRC.

The origin of circulating DPPIV in patients with cancer is a controversial issue. Current hypotheses place its origin in the liver and the immune cells [14] and in tumor microenvironment [8,14,19,38]. We performed immunohistochemical analysis throughout the adenoma-carcinoma sequence to know the location of DPPIV in the colorectal tissues. Non neoplastic cells of colorectal mucosa did not stain with DPPIV. By contrast, adenoma and CRC cells showed positive cytoplasmic immunostaining with luminal membrane reinforcement. This immunohistochemical pattern agrees with the higher activity and mRNA levels we have also found in neoplasms. Additionally, we found immunostaining with DPPIV in the inflammatory cells of lamina propria in normal and neoplastic mucosa. This finding could suggest that DPPIV is released from both immune and neoplastic cells in CRCs.

In conclusion, the present study demonstrates that DPPIV is up-regulated in adenomatous and CRC tissues when compared with the uninvolved mucosa. Plasmatic DPPIV activity is lower than in healthy subjects and is independently associated with worse 5-year survival in CRC patients. The DPPIV activity determination in the plasma is a safe, minimally invasive and inexpensive method, and may be complementary to the determination of the CD26 protein levels CRC patients. New studies with higher number of patients, collecting the preoperative and postoperative plasma samples at several time points [21], should be performed to confirm the predictive value of these results at diagnostic time and during patients’ follow-up.
